# Retroperitoneal solitary neurofibroma mimicking lymph node metastasis of colon cancer: a case report

**DOI:** 10.1186/s40792-023-01617-8

**Published:** 2023-03-27

**Authors:** Takahiro Haruna, Hideyuki Takata, Satoshi Mizutani, Akira Katsuno, Ryosuke Nakata, Norio Motoda, Nobuhiko Taniai, Hiroshi Yoshida

**Affiliations:** 1 Department of Digestive Surgery, Nippon Medical School Musashikosugi Hospital, 1-383, Kosugimachi Nakahara-Ku, Kawasaki-Shi, Kanagawa, 211-8533 Japan; 2 Department of Diagnostic Pathology, Nippon Medical School Musashikosugi Hospital, 1-383, Kosugimachi Nakahara-Ku, Kawasaki-Shi, Kanagawa, 211-8533 Japan; 3 Department of Gastrointestinal Hepato-Biliary-Pancreatic Surgery, Nippon Medical School, 1-1-5, Sendagi, Bunkyo-Ku, Tokyo, 113-8603 Japan

**Keywords:** Solitary neurofibroma, Retroperitoneal tumor, Liver metastasis, Colon cancer

## Abstract

**Background:**

A neurofibroma is a benign tumor that arises from Schwann cells and neurofibromas occur throughout the skin of neurofibromatosis type 1 (NF-1: Von Recklinghausen’s disease) patients. A retroperitoneal solitary neurofibroma without any clinical signs of NF1 has been rarely reported. Herein, we present a case of a retroperitoneal solitary neurofibroma mimicking lymph node metastasis of colon cancer as well as a literature review.

**Case presentation:**

An 80-year-old woman with abdominal pain and nausea was transported and diagnosed with bowel obstruction arising from sigmoid colon cancer A colonic stent was inserted to alleviate the bowel obstruction. A computed tomography scan with contrast revealed a liver tumor in segment 3, and an enlarged lymph node around the abdominal aorta. Whole-body 18F-fluorodeoxyglucose-positron emission tomography–CT (FDG-PET–CT) examine revealed increased FDG uptake in the liver tumor and enlarged lymph node. Liver and distant lymph node metastasis were diagnosed and we made a plan for a two-stage operation of the colon cancer and the metastatic lesions because laparotomy resection was needed for the retroperitoneal lymph node. Laparoscopic sigmoid colectomy was performed first. Pathological examination showed a tubular adenocarcinoma. A laparotomy for the metastatic lesions was performed to ensure complete lymph node dissection secondly. Histopathological findings of the liver tumor showed metastasis of sigmoid colon cancer. However, the tissue regarded as the enlarged lymph node was diagnosed as a neurofibroma. No metastasis and recurrence were observed.

**Conclusion:**

Although most neurofibromas are benign, malignant transformation of a neurofibroma is possible. PET–CT showed our patient had a high accumulated retroperitoneal tumor co-existing with colon cancer and liver metastasis. The treatment strategy of a solitary neurofibroma must be selected carefully considering the site of occurrence and the patient’s background and aggressive resection of a tumor co-existing with another malignant tumor is needed.

## Background

A neurofibroma is a benign soft-tissue tumor that arises from Schwann cells, which are cells that are found in the peripheral nervous system and form a myelin sheath. Neurofibromas have been classically associated with neurofibromatosis type I (NF-1: Von Recklinghausen’s disease). They are found in diverse anatomical locations but rarely occur in a retroperitoneal location [1]. Moreover, a solitary neurofibroma without any clinical signs of NF-1 is rarely reported and diagnosis is very difficult. A solitary neurofibroma may grow to a considerable size and cause malignant transformation infrequently. Complete surgical resection is the only treatment for these tumors. Herein, we present a patient with a retroperitoneal solitary neurofibroma mimicking lymph node metastasis of colon cancer as well as a literature review.

## Case presentation

**Fig. 1:**
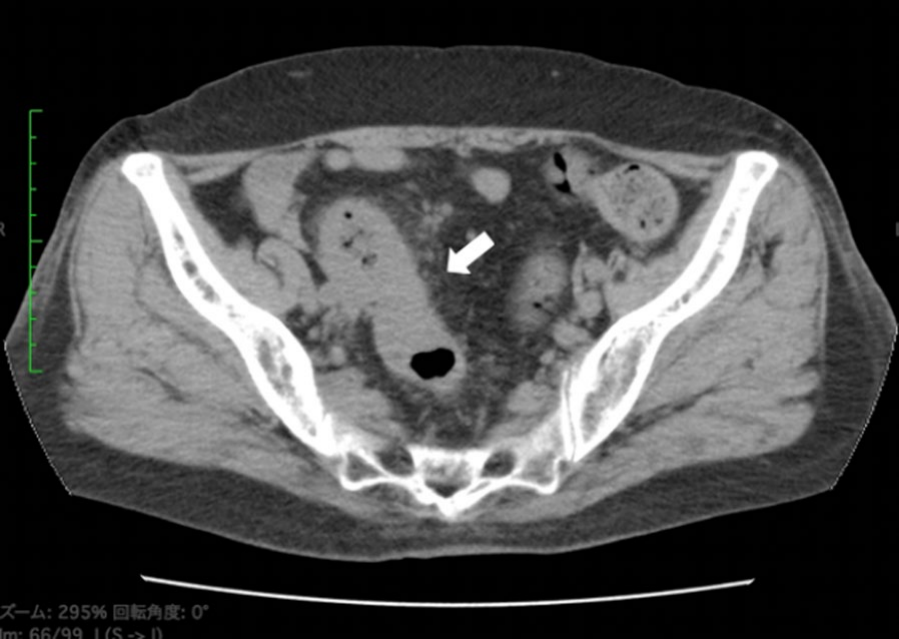
CT finding at first visit. CT scan showed increased adipose tissue concentration around sigmoid colon and thickening of sigmoid colon wall (arrow). It caused bowel obstruction and was suspected sigmoid colon cancer

**Fig. 2:**
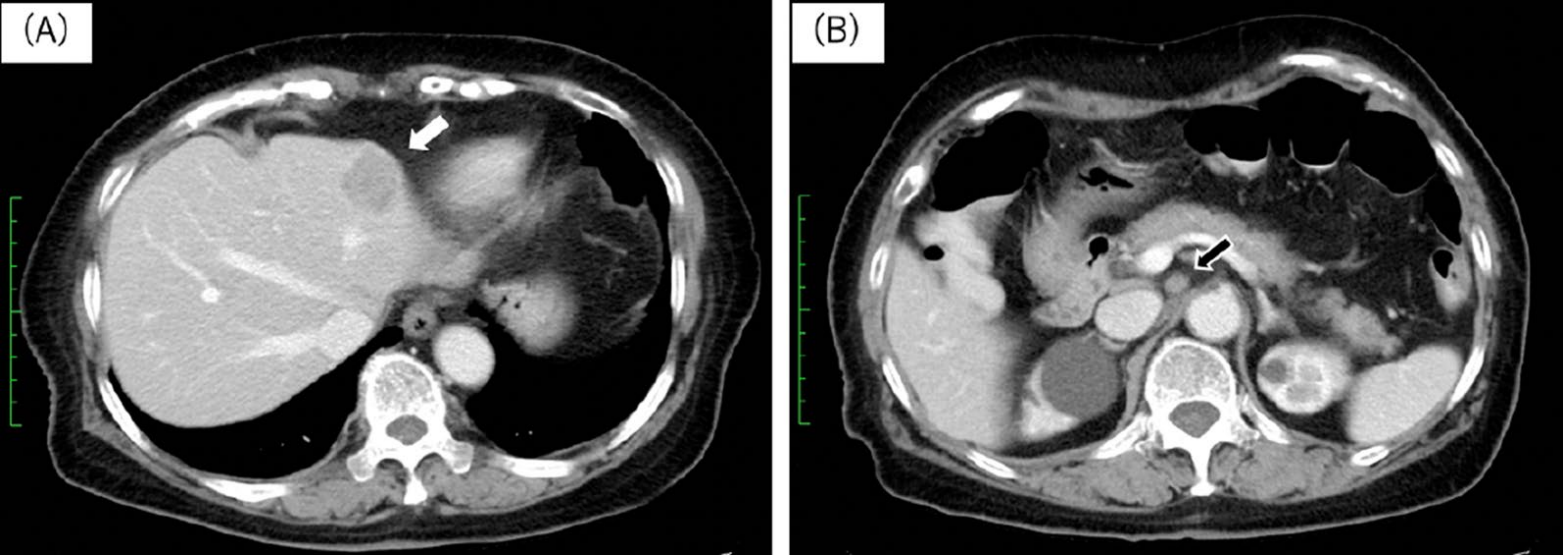
Preoperative CT findings. **A** CT scan showed a ring enhanced liver tumor (30 × 20 mm) in S3 liver lesion (white arrow). It was suspected a liver metastasis of sigmoid colon cancer. **B** CT scan showed an enlarged lymph node (10 × 10 mm) around abdominal aorta with contrast effect (black arrow). It was suspected a distant lymph node metastasis

An 80-year-old woman with abdominal pain and nausea was transported to our hospital and diagnosed with bowel obstruction arising from sigmoid colon cancer (Fig. 1). A colonic stent was inserted to alleviate the bowel obstruction. She had hypertension and dyslipidemia and a history of a previous appendectomy for acute appendectomy and hysterectomy for hysteromyoma. She had no family history of NF1 or any other genetic syndrome. A computed tomography (CT) scan with contrast revealed a liver tumor (30 × 20 mm) in segment 3, suspected metastasis of sigmoid colon cancer, and an enlarged lymph node (10 × 10 mm) around the abdominal aorta (Fig. 2). Blood test revealed an increased carcinoembryonic antigen (CEA) score (10.07 ng/ml) and normal cancer antigen 19-9 (CA19-9) score (5 ng/ml). Sigmoid colon cancer with a single liver tumor and a distant lymph node metastasis was diagnosed. We planned a subsequent resection procedure to ensure complete tumor resection. After inserting the colonic stent, her abdominal pain and nausea was disappeared, she could take a meal and was discharged to her home.

**Fig. 3:**
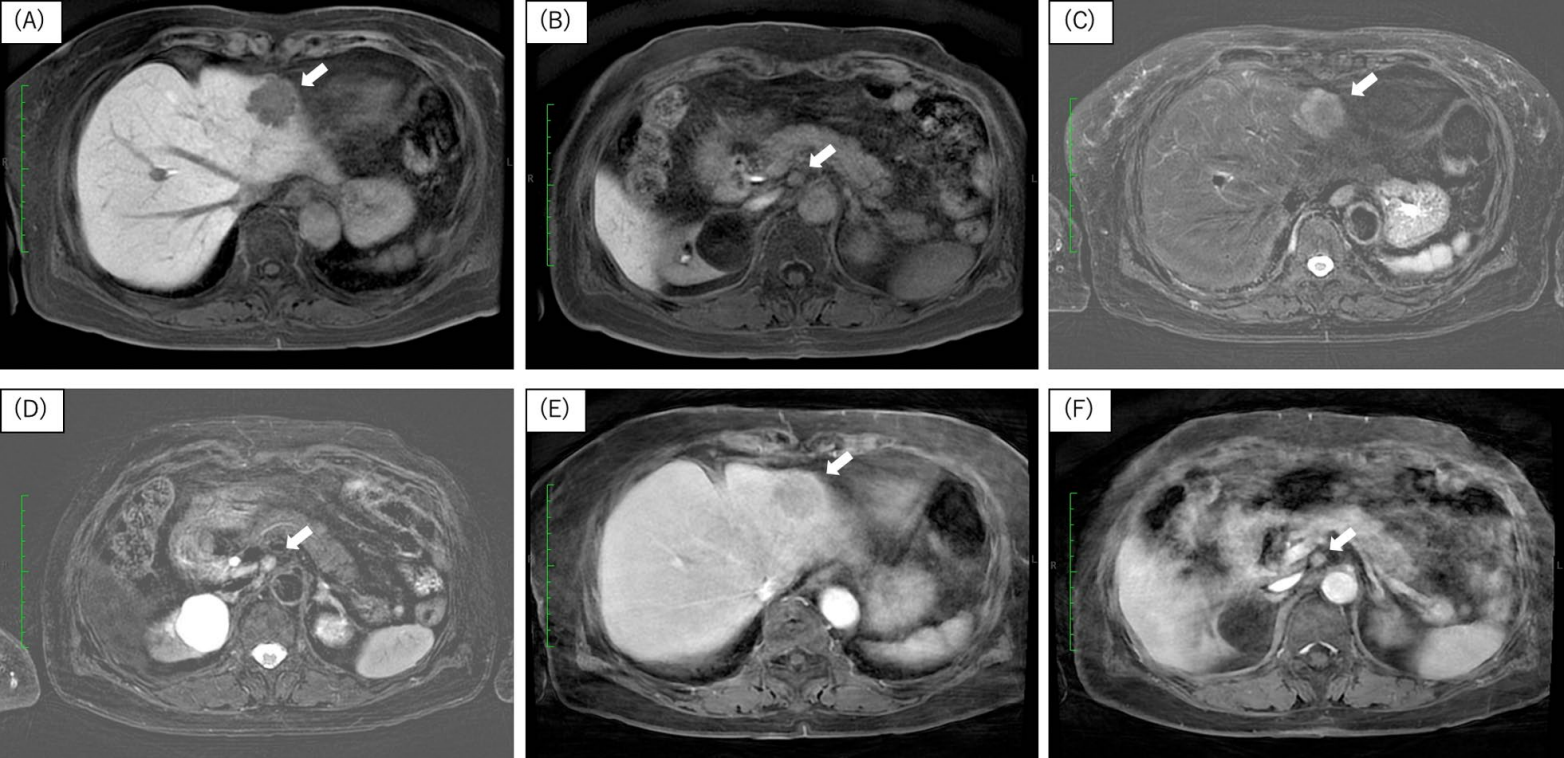
Preoperative MRI findings. **A**, **B** MRI (T1) showed the tumors as irregular margins and low signal lesions (white arrows). **C**, **D** MRI (T2) showed the liver tumor as heterogeneous high signal lesion and the retroperitoneal tumor as regular high signal lesion. **E**, **F** Contrast-enhanced MRI showed same findings as contrast-enhanced CT scan. They were suspected metastasis lesion of colon cancer

**Fig. 4:**
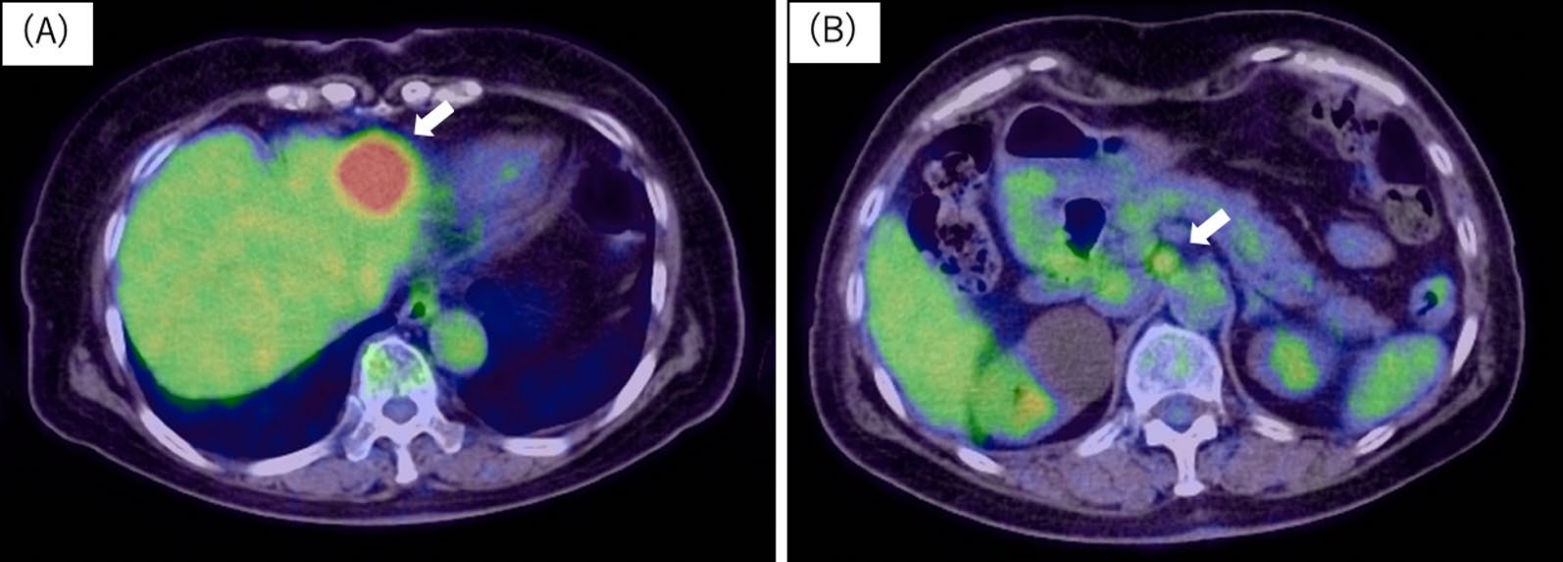
Preoperative PET–CT findings. **A**, **B** PET–CT showed the liver tumor and the retroperitoneal tumor as intense FDG uptake lesions

**Fig. 5:**
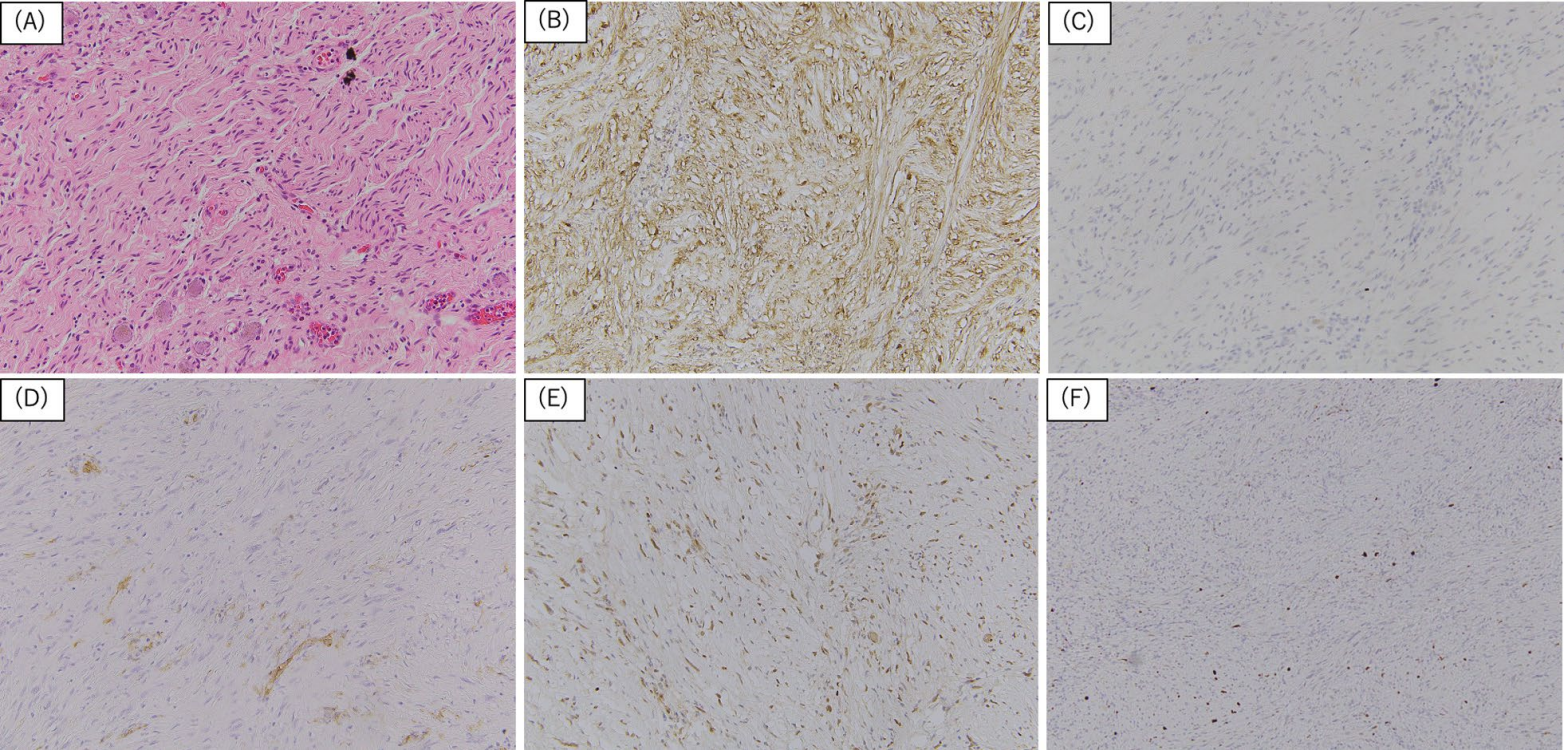
Pathological findings. **A** Hematoxylin and eosin stain showed complicated spindle cells with polymorphic nuclei and eosinophilic cytoplasm (× 200). **B** Immunohistochemical stain for S100 was positive (× 200). **C**–**E** Immunohistochemical stain for Desmin and CD34, c-kit were negative (× 200). **F** Immunohistochemical stain for MIB-1 index was 2% (× 100)

She subsequently underwent gadoxetic acid enhanced magnetic resonance imaging (EOB-MRI) and a whole-body 18F-fluorodeoxyglucose-positron emission tomography–CT (FDG-PET–CT) to search for additional liver and distant metastasis or the other primary lesion. MRI(T1) scan showed the liver tumor and enlarged lymph node had irregular margins and were low signal lesions. MRI(T2) showed the liver tumor was a heterogeneous high signal lesion and the enlarged lymph node was a regular high signal lesion. EOB-MRI showed the same findings as the contrast-enhanced CT scan and metastasis of colon cancer was confirmed (Fig. 3). Moreover, PET–CT showed the sigmoid colon cancer (SUV max: 11.2) and the liver tumor (SUV max: 10.9) and the enlarged lymph node (SUV max: 3.5) had intense FDG uptake lesions (Fig. 4). Liver and distant lymph node metastasis was confirmed and we made a plan for a two-stage operation of the colon cancer and the metastatic lesions because laparotomy resection was needed for the retroperitoneal lymph node. Laparoscopic sigmoid colectomy was performed first and the patient was discharged without any complications on the 10^th^ postoperative day. Pathological examination showed a tubular adenocarcinoma [Type 2, 65 × 20 mm, moderately differentiated adenocarcinoma, pT4(SE), L1, V1, Pn1, pN2a (4/16), M1b, pStage IVb] according to the 8th UICC classification. Next, a laparotomy for the metastatic lesions was performed to ensure complete lymph node dissection. A partial hepatectomy (S3) and lymph node dissection around the abdominal aorta was performed 1 month after the first surgery. The retroperitoneal tumor was a white and elastic, soft, and 7 × 8 mm in size. She was discharged without any complications on the 11th postoperative day. Histopathological examination of the liver tumor showed a moderately differentiated adenocarcinoma, compatible with metastasis of sigmoid colon cancer. However, the tissue regarded as the enlarged lymph node was diagnosed as a neurofibroma (S-100: positive, CD34, c-kit, desmin: negative, and Ki-67: 2%) and not colorectal cancer metastasis (Fig. 5). There are no lymph nodes and nerve tissue in the resected tissue. Moreover, it was supposed that the neurofibroma derived from a right brunch of sympathetic nerve trunk around aorta. No metastasis and recurrence have been observed for 11 months.

## Discussion

Neurofibromatosis (NF) is a genetic disorder that causes multiple tumors to appear on nerve tissues, including the brain, spinal cord, and peripheral nerves [2]. There are three types of NF: NF1, NF2, and schwannomatosis (SWN) [3]. NF1 is the most prevalent, accounting for 96% of all cases, and it is characterized by neurofibromas (peripheral nerve tumors). NF is an autosomal dominant genetic syndrome caused by mutations in genes coding for neurofibromin [4]. NF2 and SWN types are rare compared to NF1 and they occur in 3% and < 1% of the population, respectively [5]. NF1, which is known as von Recklinghausen’s disease, causes various manifestations such as multiple flat, light-brown patches of skin pigment (café-au-lait spots), skinfold freckling, visible neurofibromas under the skin, and small nodules of the iris (Lisch nodules). Diagnostic criteria for NF1 were established by the National Institutes of Health in 1988 and are listed in Table 1. Our patient did not have typical sign in the symptoms and, therefore, she was finally diagnosed with a solitary neurofibroma. A retroperitoneal solitary neurofibroma occurs rarely and usually without any symptoms. The neurofibroma in our case was found accidentally. Classic manifestations of solitary neurofibromas are not explicit and vary according to their respective locations [6]. Most are discovered as a result of their impingement on contiguous structures, which may result in palpable masses and colicky abdominal pain, and transit disorders exerting extraluminal pressure [6].

**Table 1:** NIH consensus guidelines

NIH consensus guidelines: diagnostic criteria for neurofibromatosis I
Two or more of the following
1. Six or more café-au-lait macules that are (in greatest diameter) > 5 mm in pre-pubertal individuals > 15 mm in post-pubertal individuals
2. Two or more neurofibromas of any type, or one plexiform neurofibroma
3. Axillary/inguinal freckling
4. Optic glioma
5. Two or more Lisch nodules
6. Distinctive osseous lesion (i.e., sphenoid dysplasia or thinning of long bone cortex with or without pseudoarthrosis)
7. First degree relative with NF-1

Occurrence sites of solitary neurofibromas reported in the literatures are mainly limb and trunk skin and the oral region, the retroperitoneal solitary neurofibroma is rare case. Solitary neurofibromas are solid and nonencapsulated tumors that tend to grow slowly and there is no preference for sex or age [7]. Some studies have reported rare clinical manifestations of a solitary neurofibroma. Shahid O et al. reported a 40-year-old male who presented with a 3-month history of painful calf swelling. He was later diagnosed with deep vein thrombosis that resulted from a solitary retroperitoneal neurofibroma (75 mm) impinging on the inferior vena cava [8]. Krzysztof et al. reported a 41-year-old man who complained of mild abdominal pain in the right lower quadrant. The symptom resulted from a solitary retroperitoneal neurofibroma (80 mm) between the right psoas and iliacus muscles [9].

The neurofibroma in this case was found accidentally when we performed screening for the staging of the colon cancer. The CT and MRI (T2) results showed the neurofibroma was a contrast effect lesion, and PET–CT showed the tumor was an intense FDG uptake lesion resulting from distant lymph node metastasis of the colon cancer. We employed an aggressive resection plan for the tumors.

A variety of CT findings of with solitary neurofibromas have been reported; e.g., smooth-contoured hypodense lesions with intermediate contrast enhancement or low attenuation, hypodense lesions, and heterogeneously intermediate enhancing lesions with calcification [10–12]. MRI findings also showed intermediate intensity in T1 images and high intensity with a well-circumscribed low intensity center in T2 images [10]. Similar to our case, Lariana L et al. also reported a solitary retroperitoneal tumor as an intense FDG uptake lesion and they diagnosed the tumor as a solitary neurofibroma without malignant features [13]. Solitary neurofibromas are reported as benign tumors in many cases; however, positive findings with PET–CT have suggested malignancy. PET–CT findings of solitary neurofibromas have been rarely reported and no consensus has been achieved. If solitary neurofibromas and other malignant tumors are found at same time, it is difficult to deny the relationship of the tumors with image examination only. In any case, tissue biopsy remains the gold standard for diagnosis, however, most report did not recommend diagnostic biopsy because of a risk of dissemination at the tumor biopsy site. We could not diagnose the tumor preoperatively in our patient.

Complete surgical resection is the only treatment for the tumor and patients generally have an excellent prognosis post-resection [7]. Furthermore, the tumor is basically benign tumor and a small or non-malignant tumor might be selected for follow-up depending on the occurrence site. Malignant transformation and recurrence of solitary neurofibromas have not been reported, however, Razek AAKA reported NF1 patients with malignant transformation of neurofibromas [14]. Malignant peripheral nerve sheath tumors (MPNSTs) are highly aggressive tumors and are associated with low survival rates. MPNSTs are present in 2–5% of the NF1 patients [15]. Neurofibroma histopathology shows bland spindle cell proliferation and immunohistochemical staining is positive for S100, CD34 and factor XIIIa and negative for c-kit and desmin [16]. The exact pathogenesis of a solitary neurofibroma remains unclear and genetic studies of neurofibromas in the literature are scarce. The identification of genetic aberrations in these tumors are not expected to play a role in diagnosis [17].

Retroperitoneal tumors are extremely rare tumors occurring in the retroperitoneal, ordinary develop from soft tissues including fats, muscles, nerves, lymph nodes and blood or lymphatic vessels. Approximately 70–80% of primary retroperitoneal soft-tissue tumors are malignant [18], most of the malignant tumors are retroperitoneal sarcoma [19]. Most prominent benign retroperitoneal tumors are neurogenic tumors, schwannomas are reported to be most common (0.7–2.7%) in all retroperitoneal tumors [20]. It often difficult to differentiate the most retroperitoneal tumors by image findings and there is a wide variation of the SUV max. Lim et al. reported the optimal diagnostic flowchart for retroperitoneal tumors. In a subgroup of retroperitoneal tumors with a fat component, both SUV max and tumor size were significantly different between benign and malignant retroperitoneal tumor [21]. According to the flowchart, tumors without fat component like our case are suspected benign tumors if SUV max is under 4.8. Therefore, it was possible to select only performing laparoscopic liver resection for the liver metastasis.

Recently, multimodal therapy for colon cancers with distant metastasis has progressed and aggressive resection of tumors for R0 has been performed [22]. Treatments for liver metastasis of colon cancer have become diverse due to the development of laparoscopic liver resection. In our case, we performed laparoscopic sigmoid colectomy once for obstructive colon cancer after inserting a colonic stent. Laparotomy was performed second for a single liver metastasis (S3) resection and lymph node resection around the abdominal aorta. Laparoscopic resection for the liver metastasis may have been chosen if our patient had the liver metastasis only; however, it was extremely difficult to distinguish whether the retroperitoneal tumor was benign or malignant preoperatively. Aggressive resection was needed for the retroperitoneal tumor because it co-occurred with other malignant tumors. Eleven cases of solitary retroperitoneal neurofibromas are listed in Pubmed (Table 2) [8, 9, 13, 17, 23–29] and no cases were diagnosed preoperatively. Mean age is 47.5 years old (33–73 years old) and mean tumor size is 127.5 mm (45–500 mm). The reported symptoms are diverse such as back pain and gross hematuria, cold feeling in the leg because of tumors pressing the nerves and kidney and vena cava. Similarly, the reported tumor sites are diverse same as anteromedial to the right psoas and posterior to the urinary bladder, in the adrenal gland. All cases received complete resection and no recurrences were reported. In addition, our patient had a small tumor and, as a benign tumor, observation for the tumor may have been acceptable. If we discovered the retroperitoneal solitary neurofibroma by a periodic inspection accidentally, the highly invasive operation might not have been chosen and we might have recommended observation due to no complications. The treatment strategy for a solitary neurofibroma must be selected carefully considering the occurrence site and the patient’s background. Moreover, a solitary neurofibroma is needed to be listed as one of the differential diagnoses for retroperitoneal tumors and further cases are needed to reveal the pathogenesis.

**Table 2:** The reported 11 cases of the retroperitoneal solitary neurofibroma

Author/ year	Sex	Age (y/o)	Symptom	Image study	Tumor site	Size (mm)	Preoperative diagnose	Treatment	Metastasis or recurrences
Imai H/ 1989 [23]	M	57	Back pain and microhematuria	CT	Uncinate process of the pancreas	80	Pancreatic tumor	Surgery	–
Ishikawa J/ 1989 [24]	F	56	Dysuria and left lower limb pain	CT	Retrovesical space	80	–	Surgery	None
Bastounis E/ 1997 [25]	M	40	Cold feeling in the right leg	CT, MRI	Anteromedial to the right psoas muscle	70	–	Surgery	None
Ameur A/ 2002 [26]	F	73	Flank pain	US, CT	-	220	–	Surgery	None
Corbellini C/ 2012 [27]	M	47	Abdominal pain in the right lumbar	CT	In front of the right psoas muscle	54	Neurogenic tumor (CT guided biopsy)	Surgery	None
Singh BP/ 2015[28]	M	47	Gross hematuria and left flank pain	CT	Left renal pelvis region	45	–	Surgery	None
Shen X/ 2016 [29]	F	45	Right buttock sizeable tumor	CT	Occupying the mid-abdomen	500	–	Surgery	None
Dąbkowski K / 2017 [9]	M	41	Right lower abdominal pain	Colonoscopy CT	Between the right psoas and iliacus muscle	90	–	Surgery	None
Chao/ 2018 [17]	F	33	None	CT	Posterior to the urinary bladder	69	Benign ovarian tumor	Surgery	None
Lariana L/2020 [13]	F	43	Persistent right-sided lumbar pain	CT, MRI PET–CT	In the right adrenal gland	120	Adrenal cancer	Surgery	None
Shahid O/ 2022 [8]	M	40	Painful calf swelling in the left leg	CT, MRI	Prevertebral space (L4 and L5)	75	–	Surgery	None

## Conclusion

We encountered a patient with a retroperitoneal solitary neurofibroma, which was difficult to distinguish from lymph node metastasis of sigmoid colon cancer. This type of benign tumor is encountered rarely and there have been no reports of a preoperative diagnosis. The tumor co-existing with other malignant tumors is needed resection.

## Data Availability

The dataset supporting this article is available in the Department of Digestive Surgery, Nippon Medical School Musashikosugi Hospital.

## Abbreviations

NF-1: Neurofibromatosis type I
CT: Computed tomography
CEA: Carcinoembryonic antigen
CA19-9: Cancer antigen 19-9
MRI: Magnetic resonance imaging
FDG-PET–CT: Fluorodeoxyglucose-positron emission tomography–CT
SWN: Schwannomatosis
MPNSTs: Malignant peripheral nerve sheath tumors

## References

[1] Bakhshi GD, Tayade MB, Yadav RB, Jadhav KV, Shenoy SS, Amin MV (2014). Pelvic neurofibroma. Clin Pract.

[2] Nix JS, Blakeley J, Rodriguez FJ (2020). An update on the central nervous system manifestations of neurofibromatosis type 1. Acta Neuropathol.

[3] Kresak JL, Walsh M (2016). Neurofibromatosis: a review of NF1, NF2, and Schwannomatosis. J Pediatr Genet.

[4] Ferner RE, Gutmann DH (2002). International consensus statement on malignant peripheral nerve sheath tumors in neurofibromatosis. Cancer Res.

[5] Ryouta T (2021). Current understanding of neurofibromatosis type 1, 2, and Schwannomatosis. Int J Mol Sci.

[6] Njoumi N, Elabsi M, Attolou G, Elouazzani H, Elalami FH, Chkoff MR (2015). Solitary preperitoneal neurofibroma: a case report. BMC Res Notes.

[7] Barajas-Gamboa JS, Flórez-Salamanca L (2009). Solitary neurofibroma in the abdominal wall of a patient without neurofibromatosis: case report. Biomedica.

[8] Shahid O, Khan R, Shahid M, Khan MT, Iqbal M (2022). Solitary retroperitoneal neurofibroma associated with deep vein thrombosis in a 40-year-old male. Cureus.

[9] Dąbkowski K, Marlicz W, Kaseja K, Sawicki M, Waloszczyk P, Starzyńska T (2017). Solitary retroperitoneal neurofibroma: not as small as it seems. Pol Arch Intern Med.

[10] Topsakal C, Erol FS, Ozercan I, Murat A, Gurates B (2001). Presacral solitary giant neurofibroma without neurofibromatosis type 1 presenting as pelvic mass. Case report Neurol Med Chir (Tokyo).

[11] Dafford K, Kim D, Reid N, Kline D (2007). Pelvic plexus tumors. Neurosurg Focus.

[12] Gupta P, Aggarwal R, Sarangi R (2015). Solitary neurofibroma of the adrenal gland not associated with type-1 neurofibromatosis. Urol Ann.

[13] Lariana L, Sabine B, Walter K, Bassel A, Michael W, Bernhard W (2020). A rare case of solitary retroperitoneal neurofibroma mimicking carcinoma of the adrenal gland. J Surg Case Rep.

[14] Razek AAKA (2018). MR imaging of neoplastic and non-neoplastic lesions of the brain and spine in neurofibromatosis type I. Neurol Sci.

[15] Deger AN, Bayar MA, Caydere M, Deger H, Tayfur M (2015). Retroperitoneal malignant peripheral nerve sheath tumour: a rare case report. J Clin Diagn Res.

[16] Hechtman JF, Harpaz N (2015). Neurogenic polyps of the gastrointestinal tract: a clinicopathologic review with emphasis on differential diagnosis and syndromic associations. Arch Pathol Lab Med.

[17] Chao WT, Liu CH, Chen YJ, Wu HH, Chuang CM, Wang PH (2018). Neurofibroma involving obturator nerve mimicking an adnexal mass: a rare case report and PRISMA-driven systematic review. J Ovarian Res.

[18] Neville A, Herts BR (2004). CT characteristics of primary retroperitoneal neoplasms. Crit Rev Comput Tomogr.

[19] Rosenberg AE (2013). WHO classification of soft tissue and bone, fourth edition: summary and commentary. Curr Opin Oncol.

[20] Li Q, Gao C, Juji JT, Hao X (2007). Analysis of 82 cases of retroperitoneal schwannoma. ANZ J Surg.

[21] Lim CH, Seok HY, Hyun SH, Moon SH, Cho YS, Lee KH, Kim BT, Choi JY (2019). Evaluation of a diagnostic 18F-FDG PET/CT strategy for differentiating benign from malignant retroperitoneal soft-tissue masses. Clin Radiol..

[22] Shinji S, Yamada T, Matsuda A, Sonoda H, Ohta R, Iwai T, Takeda K, Yonaga K, Masuda Y, Yoshida H (2022). Recent advances in the treatment of colorectal cancer: a review. J Nippon Med Sch.

[23] Imai H, Kobayashi J, Manabe R, Namio H, Ichinona T (1989). A case of neurofibroma located in the retroperitoneum involving the uncinate process of the pancreas. Gastroenterol Jpn.

[24] Ishikawa J, Kamidono S, Maeda S, Sugiyama T, Hara I, Takechi Y (1989). Solitary retroperitoneal neurofibroma: a case report. Acta Urol Jpn.

[25] Bastounis E, Asimacopoulos PJ, Pikoulis E, Leppaniemi AK, Aggouras D, Papakonstadinou K, Papalambros E (1997). Benign retroperitoneal neural sheath tumors in patients without von Recklinghausen’s disease. Scand J Urol Nephrol.

[26] Ameur A, Lezrek M, Jira H, el Alami M, Beddouch A, Abbar M (2002). Solitary giant retroperitoneal neurofibroma. Prog Urol.

[27] Corbellini C, Vingiani A, Maffini F, Chiappa A, Bertani E, Andoreoni B (2012). Retroperitoneal pararenal isolated neurofibroma: report of a case and review of literature. Ecancemedicalscience.

[28] Singh BP, Krishnaswamy SA, Singhai A, Sankhwar S (2015). Parapelvic solitary neurofibroma of the kidney. BMJ Case Rep..

[29] Shen XQ, Shen H, Wu SC, Lv Y, Lu H, Lin XJ (2016). Surgically treated solitary giant gluteal and retroperitoneal neurofibroma: a case report. World J Surg Oncol.

